# Rasch modelling to deal with changes in the questionnaires used during long-term follow-up of cohort studies: a simulation study

**DOI:** 10.1186/s12874-016-0211-6

**Published:** 2016-08-24

**Authors:** Alexandra Rouquette, Sylvana M. Côté, Jean-Benoit Hardouin, Bruno Falissard

**Affiliations:** 1 CESP, INSERM, Univ. Paris-Sud, UVSQ, Université Paris-Saclay, U1178, Maison de Solenn, 97 boulevard du Port-Royal, 75014 Paris, France; 2 Biostatistics and Epidemiology Department, AP-HP, Hôtel-Dieu Hospital, Université Paris Descartes, 1 place du parvis Notre-Dame, Paris, 75004 France; 3 Research Unit on Children’s Psychosocial Maladjustment, University of Montreal, 3050 Edouard Montpetit, Montreal, H3T 1 J7 QC Canada; 4 Department of preventive and social medicine, University of Montreal, C.P. 6128, succursale centreville, Montreal, H3C 3 J7 Québec Canada; 5 EA 4275-SPHERE, University of Nantes, BP 53508, 1 rue Gaston Veil, Nantes, 44035 France; 6 Biostatistics and Methodology Unit, CHU de Nantes, 1 Place Alexis-Ricordeau, 44000 Nantes, France; 7 Department of Public Health, AP-HP, Paul Brousse Hospital, 12 Avenue Paul Vaillant Couturier, 94800 Villejuif, France

**Keywords:** Cohort, Longitudinal, Questionnaire, Trajectories, Score, Rasch model, Latent variable

## Abstract

**Background:**

A specific measurement issue often occurs in cohort studies with long-term follow-up: the substitution of the classic instruments used to assess one or several factors or outcomes studied by new, more reliable, more accurate or more convenient instruments. This study aimed to compare three techniques to deal with this issue when the substituted instrument is a questionnaire measuring a subjective phenomenon: one using only the items shared by the different questionnaires over time, i.e. computation of the raw score; the two others using every item, i.e. computation of the standardised score or estimation of the latent variable score using the Rasch model.

**Methods:**

Two hundred databases were simulated, corresponding to longitudinal 10-item questionnaire data from three trajectory groups of subjects for the subjective phenomenon of interest (“increasing”, “stable-low” or “stable-high” mean trajectory over time). Three copies of these databases were generated and the subjects’ responses to some items were removed at some collection times leading to a number of shared items over time varying from 4 to 10 in the 800 datasets. The performances of Latent Class Growth Analysis (LCGA) applied to the raw score, the standardised score or the latent variable score were studied on these databases according to the number of shared items over time.

**Results:**

Surprisingly, LCGA applied to the latent variable score estimate did not perform as well as LCGA applied to the standardised score, where it was the most efficient whatever the number of shared items. However, the proportions of correctly classified subjects by LCGA applied to the latent variable score were more balanced across trajectory groups.

**Conclusions:**

The use of the standardised score to deal with questionnaire changes over time was more efficient than the raw score and also, surprisingly, than the latent variable score. LCGA applied to the raw score was the least efficient and exhibited the most unbalanced misclassifications across trajectory groups. As prospective longitudinal studies with long-term follow-up are more and more common, researchers should be aware of this phenomenon and should reconsider the use of the raw score when changes in the questionnaires used occurred during follow-up.

## Background

Prospective longitudinal studies with long-term follow-up (exceeding several decades) are more and more common, since numerous cohort studies undertaken during the second half of the 20th century are still ongoing. A specific measurement issue often occurs when the follow-up is so long: the substitution of the classic instruments used to assess one or several of the factors or outcomes studied at each data collection time by new, more reliable, more accurate or more convenient instruments. This issue is of particular concern when the substitution concerns a questionnaire, i.e. an instrument assessing a subjective phenomenon (anxiety, quality of life, etc.). Indeed, in this case, the score, which is the (sometimes weighted) sum of the patient’s responses to the questionnaire items, is classically used as the measure of the subjective concept. Thus, if the questionnaire changes during follow-up, the scale on which the measure is performed also changes and the scores collected over time on these different questionnaires are no longer comparable while supposed to measure the same phenomenon.

In some longitudinal studies, a change of questionnaire is sometimes required by the population or situation under study. As an example, developmental epidemiology is an approach that incorporates the principles, theories and methods of developmental psychology into epidemiological research to explore the mechanisms by which developmental processes affect the risk of occurrence of health problems [1, 2]. Data from cohorts followed over developmental stages such as infancy, childhood, adolescence, etc., are thus required to study these processes. However, numerous subjective phenomena are differently expressed at these different stages. For example, in childhood, irritability and somatic complaints are symptoms of depression while substance abuse or hypersomnia can be manifestations of depression in adolescence [3]. An adaptation of the questionnaire is thus needed during the follow-up of the cohort, with some items dropped, added or modified, to assess the same phenomenon depending on the developmental stage.

**Fig. 1:**
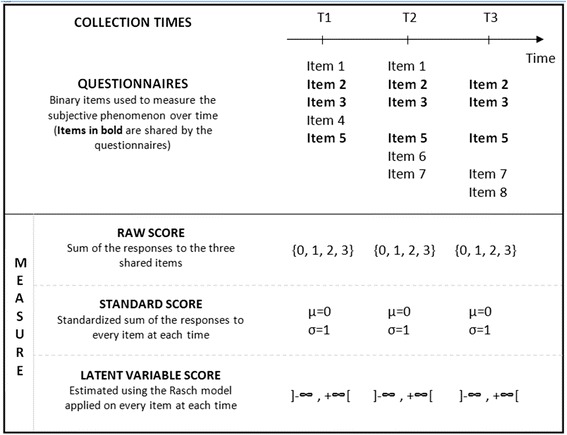
The three techniques used to obtain a comparable measure over time from different questionnaires. μ: mean, σ: standard deviation

In such situations, two main techniques are used in practice to obtain a measure of the subjective phenomenon that is comparable over time. In the upper part of Fig. 1, an example of a longitudinal study with three collection times is given. Different questionnaires, sharing certain items, are used to measure the subjective phenomenon under study over time. The first main technique stem from the classical test theory in which the studied construct is defined by the items used to measure it [4]. The raw score, computed using only the items that are present at every data collection time (bold items), is thus used as the measure of the subjective phenomenon. In this way, the measure is comparable over time but it can suffer from a loss of precision as the information provided by the items that are not present at every data collection time is not considered. The other main technique is inspired by the modern test theory in which a latent variable is used to represent the studied construct in the measurement model underlying the questionnaire [5, 6]. Therefore, in this theory, even if the latent variable (depression for example) is measured using different sets of items (different questionnaires), its signification remains the same (it still represents the phenomenon “depression” which is defined independently of the instrument used to measure it). In practice, it is thus the standardised score which is computed: for each individual, the raw score is calculated using all the items present at every time and is standardised using the sample mean and standard deviation at each collection time. In this case, the information provided by every item at every time is used, but as the sample mean is set at zero at each collection time, a hypothesis is implicitly made: that of the stability of the mean level of the subjective phenomenon in the sample over time. Therefore, both the raw score and the standardised score could lead to a loss of power or to biased results when used in statistical analyses.

The Rasch model is a latent variable model which expresses the probability that an individual will respond positively to a binary item as a function of his/her level on the interval scale of the latent variable and of an item parameter termed “item difficulty” [7, 8]. One of its interesting properties, the specific objectivity, implies that, apart from sampling error, the estimations of the item parameters are invariant whatever the sample or situation studied. Similarly, apart from sampling error, the estimation of the subject’s level on the latent variable (termed “latent variable score” afterward) is identical whatever the set of items used to measure it [9]. Thus, in the situation represented in Fig. 1, the Rasch model could be used on different sets of items at each collection time to estimate the individuals’ latent variable score which would be longitudinally comparable, since the scale of the latent variable is identical at every time, provided that some shared items enable this scale to be calibrated (i.e. enabling the zero to be set at the same level on the scale over time).

The hypothesis underpinning this work is that, when questionnaire changes have occurred during the follow-up of a longitudinal study, the use of the latent variable score estimated by the Rasch model in statistical analysis could provide estimates with smaller variance and lesser bias than those obtained from a raw score or a standardised score, particularly when the number of items shared by the questionnaires over time (used to compute the raw score) is small compared to the number of items available to estimate the latent variable score at each time.

The aim of this simulation study was thus to compare the performances of a statistical method for the analysis of longitudinal data applied to the raw score, to the standardised score and to the latent variable score, according to the number of shared items across questionnaires over time. Latent Class Growth Analysis (LCGA) was chosen as the statistical method for analysis of longitudinal data to test this hypothesis, since it is a widely used method in developmental epidemiology and is also increasingly used in other fields of epidemiology [10–13]. Indeed it enables clusters of subjects with homogenous trajectories concerning the subjective phenomenon studied over time to be identified, and the associations of these clusters with a specific outcome or environmental, biological, demographic, or other factors to be evaluated [14–19].

## Methods

**Fig. 2:**
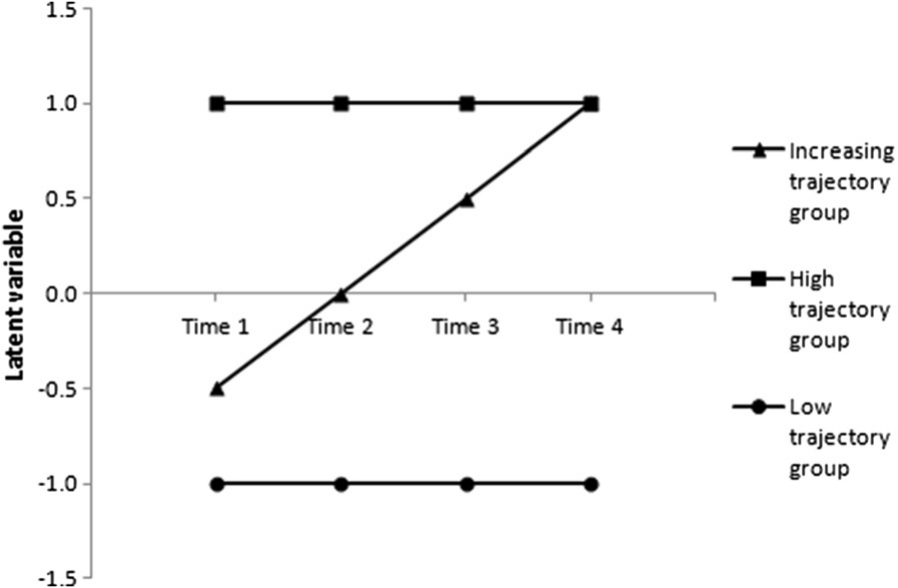
Mean trajectories for the subjective phenomenon in the three simulated groups

The scenario used to simulate data was chosen to tally with typical epidemiological studies in which the longitudinal course of a unidimensional construct is under study: a cohort study with four collection times and the same ten binary items used to assess the subjective phenomenon at each time. A mixture of three groups of equal size constituted the simulated cohort (Fig. 2): the “low” group with a low level of the subjective phenomenon over time and stable mean trajectory, the “high” group with a high level and stable mean trajectory and the “increasing” group with an increasing trajectory over time. The performances of LCGA were appraised on its ability to determine the trajectory group to which each subject belonged in the simulated datasets.

### Data generation

A longitudinal Rasch model was used to simulate the data. In this model, the probability of a positive response of subject *i* (*i* = 1 … *N*) to binary item *j* (*j* = 1 … *J*) at time *t* (*t* = 1 … *T*) is a function of his level on the latent variable at each time *t*, (*θ*_*i*_^(*t*)^), and of item difficulty (*δ*_*j*_):

$$ P\left({Y}_{ij}^{(t)}=1\Big|{\theta}_i^{(t)},\ {\delta}_j\right)=\frac{ \exp \left({\theta}_i^{(t)}-{\delta}_j\right)}{1+ \exp \left({\theta}_i^{(t)}-{\delta}_j\right)} $$

The distribution of *θ*^(*t*)^ is assumed to be a multivariate normal distribution with *μ*^(*t*)^ and *σ*^(*t*)^ being respectively the mean and the standard deviation of the latent variable in the sample at time *t*, and *σ*^(*tt* ')^ the covariance between *θ*^(*t*)^ and *θ*^(*t* ')^ with *t* ≠ *t* '. In this study, item difficulty *δ*_*j*_ was assumed to be constant over time, i.e. the longitudinal invariance of the measurement scale was hypothesized [20].

### Parameters of the simulation model

The number of collection times *T* was thus set at 4 and the number of items *J* at 10. To simulate the three different trajectory groups, three simulation models were used, each with different values for *μ*^(*t*)^ : *μ*^(*t*)^ = − 1 and *μ*^(*t*)^ = 1 irrespective of *t* in the “low” and “high” groups respectively, and *μ*^(1)^ = − 0.5; *μ*^(2)^ = 0; *μ*^(3)^ = 0.5 and *μ*^(4)^ = 1 in the “increasing” group. The size of each group was set at 1000 subjects, giving a total sample of 3000 subjects.

The latent variable variances *σ²*^(*t*)^ and covariances *σ*^(*tt* ')^ were assumed to be equal in the three groups. A LCGA on the ${\widehat{\theta}}_i$ estimated by the Rasch model was applied to data from the Quebec Longitudinal Study of Kindergarten Children (QLSKC) to set the values of *σ²*^(*t*)^ and *σ*^(*tt* ')^ in the simulation model close to those found in some real data [21]. In this cohort, internalized symptoms (mainly anxiety and depression symptoms) exhibited by 2000 children representative of the Quebec (Canada) population were longitudinally evaluated using nine items administered to their mother at every time of data collection during childhood (i.e. at ages 6, 8, 10 and 12 years). On the basis of the values found on these real data, it was decided, in the simulation model, to set *σ²*^(*t*)^ at 0.3 at each collection time, the correlations between two adjacent times at 0.8, the correlation between the 1st and 3rd time at 0.7 as well as the correlation between the 2nd and 4th time, and finally, the correlation between the 1st and the 4th time at 0.6. Item difficulty values, *δ*_*j*_, were chosen as percentiles of a normal distribution $\mathcal{N}\left(0,1\right)$.

### Analyses of the simulated databases

Once the parameters of the models were set, a series of databases was simulated. For each of the 3000 subjects, each database contained: responses to the ten binary items at each collection time, simulated trajectory group membership (“low”, “high” and “increasing”) and the simulated values for the latent variable (*θ*_*sim*_^(*t*)^) which was used in the longitudinal Rasch model to predict the responses of each subject to the ten items at each time *t*. Eight variables were added to each of the databases: the raw score (*S*^(*t*)^, the simple sum of item responses) and the standardised score (*stS*^(*t*)^, the standardised sum of item responses) at each time *t*.

**Fig. 3:**
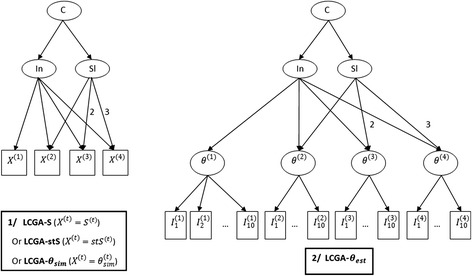
Path diagrams for the LCGA applied to the various measures of the subjective phenomenon. S^(t)^ raw score, stS^(t)^: standardised score, θ_sim_^(t)^: simulated latent variable, θ_est_: latent variable score, θ^(t)^: latent variable score estimated by the Rasch model applied to the items (I_j_^(t)^) at each time t. Factor loadings are set at 1 unless otherwise stated. In: intercept of the latent trajectory, Sl: slope of the latent trajectory, C: the latent class, LCGA: Latent Class Growth Analysis

On each of the databases, LCGA, with the number of classes set at 3 and a linear shape imposed on the latent trajectory, was consecutively applied to the variables *S*^(*t*)^ (LCGA-S), *stS*^(*t*)^ (LCGA-stS) and *θ*_*sim*_^(*t*)^ (LCGA- *θ*_*sim*_) (left part of Fig. 3). The purpose of the LCGA- *θ*_*sim*_, which would not be possible on real data, was to provide a benchmark for the best performances of LCGA, i.e. applied to the “true” (without measurement error) level on the latent variable of the subjects. Finally, LCGA (with 3 classes and a linear shape) was applied to the latent variable score (*θ*^(*t*)^) estimated by the Rasch model using the responses to the items (*I*_*j*_^(*t*)^) at each time t (LCGA- *θ*_*est*_) with an equality constraint on the item parameters across collection times (right part of Fig. 3).

### Performance criteria

The highest probability of group membership provided by the LCGA was used to assign each subject to a trajectory group. Three performance criteria were computed for each kind of LCGA: 1/ the mean proportion of correctly classified subjects across the series of simulated datasets, with the simulated trajectory group taken as reference, 2/ the kappa coefficient assessing the agreement between the trajectory group assigned by the LCGA and the simulated trajectory group (poor to moderate agreement if <0.6, substantial agreement if between 0.6 and 0.8, almost perfect agreement if >0.8) [22, 23], 3/ the mean relative entropy over the series of simulated datasets which is an index measuring the overall certainty of the classification by LCGA (i.e. the degree of separation between the trajectory groups) ranging from 0 to 1 with 1 the highest level of certainty [10, 24].

### Scenarios of items shared by questionnaires over time

**Fig. 4:**
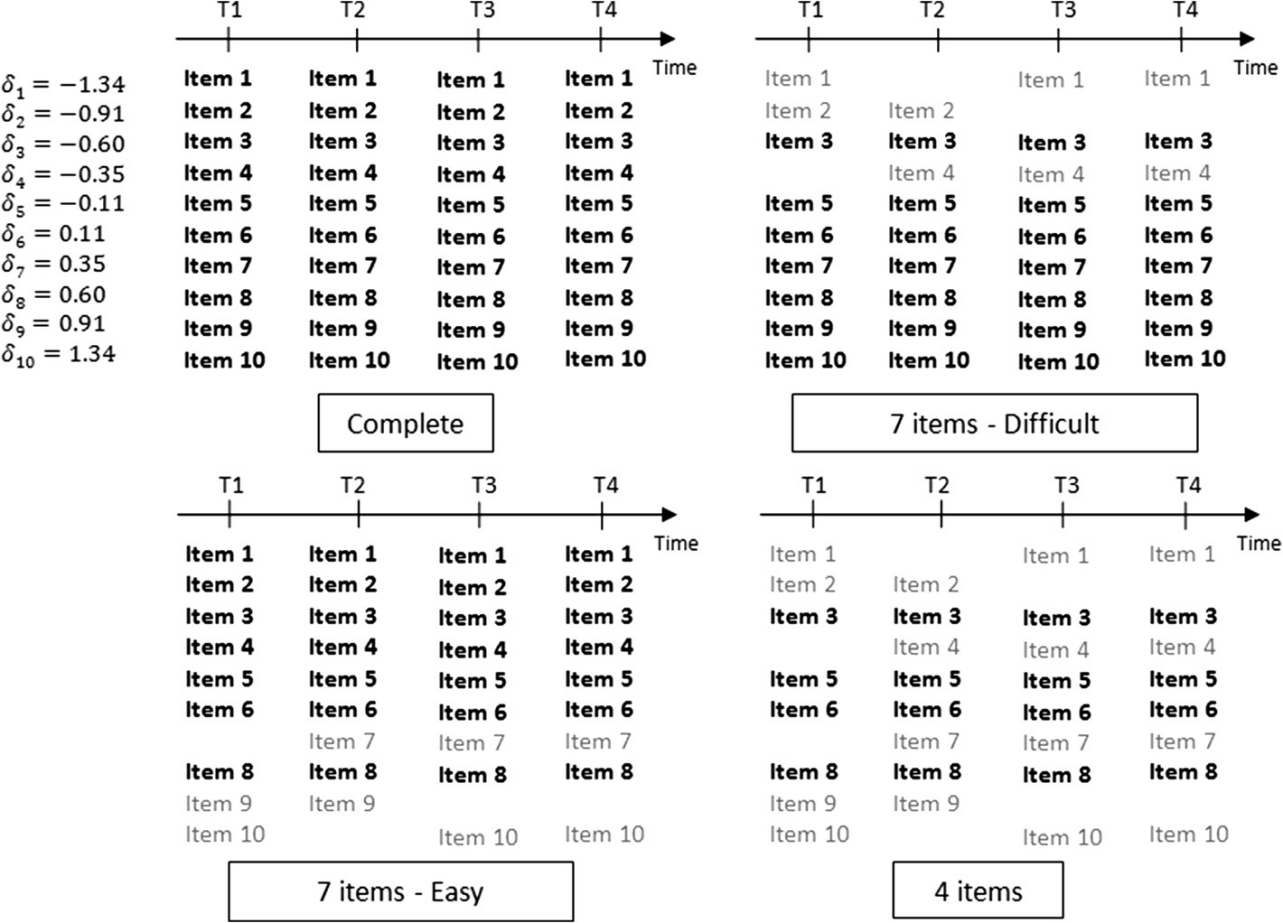
The four scenarios for items shared by the questionnaires across time. Bold items are those available to calculate the raw score, grey items are additional items available to compute the standardised score and to estimate the latent variable score. δ_j_: value for the difficulty of item j as set in the simulation model

The performances of the LCGA-S, LCGA-stS and LCGA- *θ*_*est*_ were studied in four scenarios concerning the items in common across questionnaires over time (Fig. 4). In order to do this, the series of simulated databases was duplicated to obtain four identical series. A first series was used for the “Complete” scenario in which all 10 items were available to compute the raw score, the standardised score and to estimate the latent variable score at every collection time. In a second series, data from three items with a low level of difficulty were erased in the databases at some of the collection times arbitrarily chosen (items 1, 2 and 4 in grey in the Fig. 4). While the standardised score and the latent variable score could be estimated using the items 1, 2 and 4 available at only some collection times, only 7 fairly difficult items (bold items in Fig. 4, *δ*_*j*_ *ϵ* [−0.60 ; 1.34]) were available to compute the raw score in this second series of simulated databases; this scenario was thus called the “7 items – Difficult” scenario. A third duplicated series of simulated databases was used for the “7 items - Easy” scenario which mirrored the previous one, with only seven items with a low level of difficulty (*δ*_*j*_ *ϵ* [−1.34 ; 0.60]) available to compute the raw score. Finally, in the last duplicated series, data from the same items at the same collection times as in both previous scenarios were erased. It left only four items available to compute the raw score and this last scenario was thus called the “4 items” scenario.

The raw score and the standardised score were computed afresh in the databases corresponding to the “7-items – Difficult”, “7 items – Easy” and “4 items” scenarios. Then, LCGA was applied to the raw score, to the standardised score and to the latent variable score estimated using a Rasch model on every database.

### Number of simulated datasets

On a preliminary collection of 100 simulated datasets, the standard deviations were estimated at 0.011, 0.016 and 0.009 for the proportion of correctly classified subjects, the kappa coefficient and the entropy respectively. In total, 200 datasets were therefore simulated to yield an accuracy of ±0.15 %, ±0.002 and ±0.001 for these three performance criteria respectively (type 1 error risk set at 5 %).

### Software

Stata^©^ v.12 (StataCorp LP. College Station, TX) was used to simulate the data (simirt program). Mplus^©^ v.7 (Muthen & Muthen, Los Angeles, CA) was used to estimate the latent variable score by the Rasch model using the robust maximum likelihood (MLR) estimator and to apply LCGA. The MplusAutomation package in R v.3.1.0 (R Foundation for statistical Computing, Vienna, Austria) was used to automate the application of LCGA to each of the 800 datasets and the collection of the results [25–28].

## Results

Table 1 shows the performance criteria concerning LCGA-S, LCGA-stS, LCGA- *θ*_*est*_ and LCGA- *θ*_*sim*_ according to the scenarios studied. The mean proportion of correctly classified subjects when LCGA was directly applied to *θ*_*sim*_ was 82.2 % [82.1–82.4]. This proportion was never reached when LCGA was applied to the three different measures of the subjective phenomenon estimated from item responses (S, stS and *θ*_*est*_), meaning they were affected by measurement error. When 10 items were available to compute these three measures, the mean proportion of correctly classified subjects was higher for LCGA-S and LCGA-stS, 77.4 % [77.2–77.6] and 77.5 % [77.3–77.7] respectively, than for LCGA- *θ*_*est*_: 75.6 % [75.4–75.8]. When the number of items shared by questionnaires over time decreased, the mean proportion of correctly classified subjects decreased for the three different scores, but a more marked decrease was observed for LCGA-S. Also, the mean proportion of correctly classified subjects appeared lower when the shared items had on average a lower level of difficulty, particularly in the case of LCGA-S. Finally, the LCGA-stS generally had a higher mean proportion of correctly classified subjects than LCGA-S and LCGA- *θ*_*est*_.

**Table 1:** Performance criteria of the LCGA applied to the various measures of the subjective phenomenon

Criterion	Scenario	LCGA-S	LCGA-stS	LCGA- *θ*_*est*_	LCGA- *θ*_*sim*_
%CC	Complete	77.5 [77.3 – 77.6]	77.6 [77.4 – 77.7]	75.7 [75.5 – 75.8]	82.3 [82.2 – 82.4]
	7 items - Difficult	76.3 [76.1 – 76.4]	77.2 [77.1 – 77.3]	75.1 [74.9 – 75.2]	
	7 items - Easy	73.7 [73.6 – 73.9]	76.7 [76.6 – 76.8]	75.0 [74.8 – 75.1]	
	4 items	72.8 [72.7 – 72.9]	76.3 [76.2 – 76.5]	74.3 [74.1 – 74,4]	
Kappa	Complete	0.662 [0.660 –0.664]	0.664 [0.661 –0.666]	0.635 [0.633 –0.637]	0.734 [0.732 – 0.736]
	7 items - Difficult	0.644 [0.642 –0.646]	0.658 [0.656 –0.660]	0.626 [0.624 –0.628]	
	7 items - Easy	0.606 [0.603 –0.609]	0.651 [0.649 –0.653]	0.624 [0.622 –0.627]	
	4 items	0.592 [0.590 –0.594]	0.645 [0.643 –0.647]	0.614 [0.612 –0.616]	
Entropy	Complete	0.775 [0.774 –0.776]	0.775 [0.774 –0.776]	0.755 [0.754 –0.756]	0.909 [0.909 – 0.910]
	7 items - Difficult	0.734 [0.733 –0.736]	0.761 [0.760 –0.762]	0.741 [0.740 –0.742]	
	7 items - Easy	0.730 [0.729 –0.732]	0.761 [0.760 –0.762]	0.740 [0.738 –0.741]	
	4 items	0.655 [0.653 –0.657]	0.744 [0.743 –0.745]	0.723 [0.721 –0.724]	

*Legend:* %CC: mean proportion of correctly classified subjects, *LCGA:* latent class growth analysis, *S:* raw score, *stS:* standardised score, *θ*_*est*_: latent variable score estimated by the Rasch model, *θ*_*sim*_: simulated latent variable, [95 % confidence interval]

The same pattern was observed for the two other performance criteria: the kappa coefficient and entropy. Overall, substantial agreement was found except for LCGA-S in the “4 items” scenario. For both kappa coefficient and entropy, values decreased with the number of shared items, particularly for LCGA-S, and highest values were observed for LCGA-stS.

**Fig. 5:**
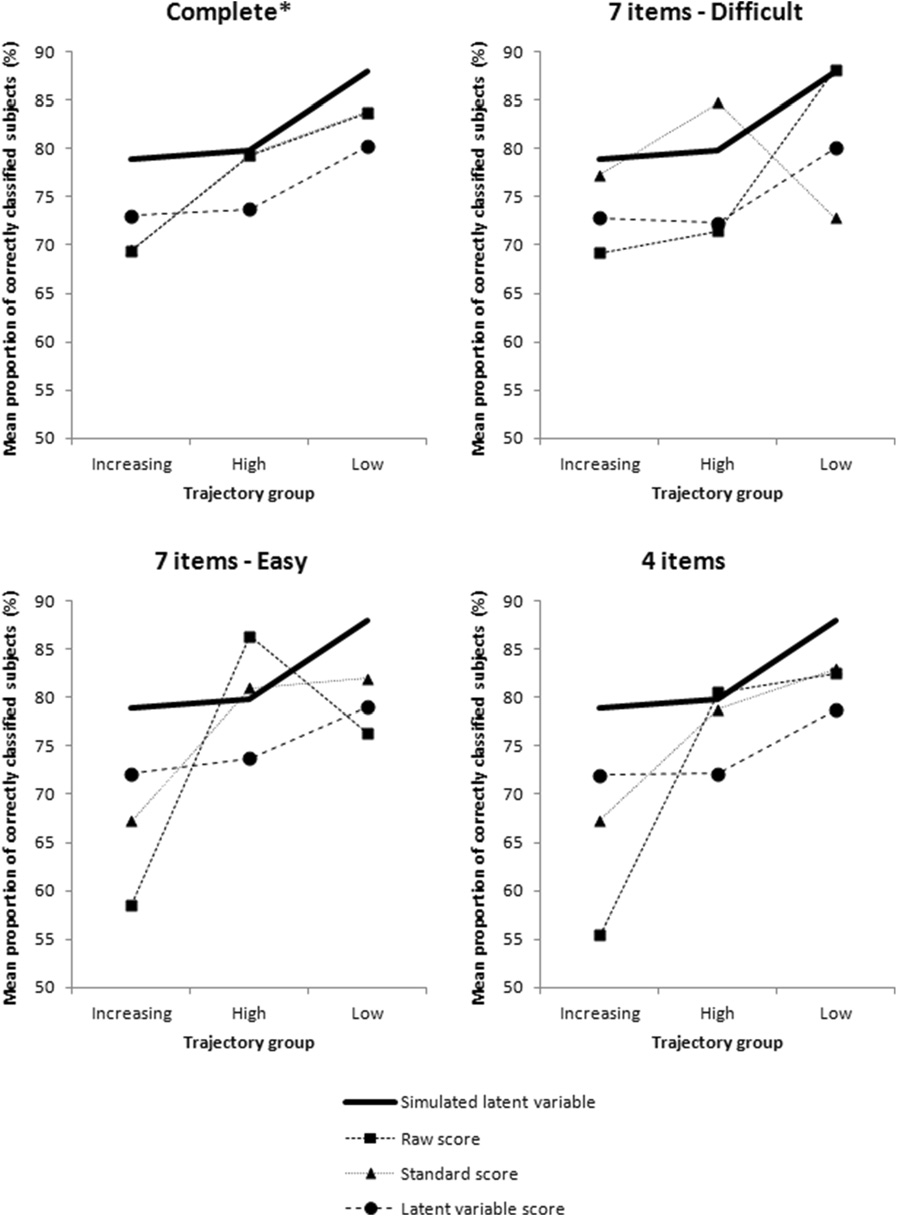
Mean proportion of correctly classified subjects according to the trajectory group for each scenario studied. * The raw score and the standardised score are superimposed in the complete scenario

In an attempt to clarify these results, the mean proportion of correctly classified subjects was studied according to trajectory group for each kind of LCGA and each scenario studied (Fig. 5). It can be observed that these proportions were quite stable (72 %–81 %) when LCGA was applied to latent variable score (LCGA- *θ*_*est*_), whatever the scenario of shared items. This was not the case for LCGA-S and LCGA-stS. Indeed, in these two cases, the proportion varied according to the scenario with fairly high values for the “high” and “low” trajectory groups, but low values (55 %–70 %) for the “increasing” group.

## Discussion

This study aimed to compare the performances of LCGA applied to the raw score, to the standardised score and to the latent variable score estimated by the Rasch model on longitudinal questionnaire data representative of data from cohort studies, particularly in the case when questionnaire changes occurred during follow-up. Surprisingly, LCGA applied to the standardised score had the highest performance criteria especially when the number of shared items by the questionnaires over time decreased. Nevertheless, LCGA applied to the latent variable score was more efficient than LCGA applied to the raw score when the number of shared items was small or their level of difficulty was low. Moreover, whatever the score (raw, standardised or latent variable) on which is applied LCGA, its performances decreased along with the number of items available to compute or estimate these scores. This was expected as the precision of the estimation of the subjective phenomenon also decrease with the number of items used to measure it.

The lower performances of LCGA applied to the latent variable score go against our hypotheses. This is surprising, especially because the simulated data on which these analyses were run were produced using the Rasch model. In practice, when a measurement scale is validated using the Rasch model, the accuracy of the raw score is higher than that of the latent variable score estimated using the Rasch model, particularly if item difficulty is to be concurrently estimated. This was the case in the present study, since we wanted to evaluate the performances of LCGA applied to the latent variable score in the same conditions as it would perform in practice. However, if the value for item difficulty would have been set in the Rasch model rather than estimated, the performances of LCGA applied to the latent variable score would probably have been higher.

When the mean proportion of correctly classified subjects was studied according to trajectory group, a lower mean proportion was observed for the “increasing group” when LCGA was applied to the raw score or standardised score than when it was applied to the latent variable score, particularly when the number of shared items was small. This can be explained, in the case of the standardised score, by the implicit hypothesis of no longitudinal mean change in the subjective phenomenon over time, which is made when the score is standardised at each collection time. In the case of the raw score, when the number of shared items decreases, the accuracy of the score is probably too low to detect a change over time. The stability of the mean proportion of correctly classified subjects over the three groups observed when LCGA was applied on the latent variable score is a property that could be interesting in epidemiological studies in which the classification into trajectory groups is often used as an outcome or an exposure factor. Misclassification on these variables can lead to information bias, and it has been shown that, when this occurs on dichotomous variables, the resulting information bias is non-differential [29]. However, this is not true for polytomous variables, and a misclassification of this sort can lead to differential information bias [30]. Further studies should be performed to determine the influence of the stability of the proportion of misclassifications across categories of polytomous variables on the resulting bias.

As with any simulation study, one criticism concerning these results is that the simulated scenarios do not cover all the possible scenarios that can be found in practice, and that these results are not necessarily transposable to every situation. However, this is, to our knowledge, the first study to have explored the influence of using the raw score or the standardised score rather than the latent variable score on the performance of statistical methods for longitudinal data. Yet this is an issue very commonly faced by epidemiologists. The influence of other parameters should be studied in further studies, such as the number of data collection times, sample size, etc. The influence of certain hypotheses in the simulation model should also be studied, such as the hypotheses specific to the Rasch model since there are questionnaires used in epidemiology that have not been validated using this model. Concerning the statistical method for the analysis of longitudinal data chosen (the LCGA), the number of groups, group size, trajectory shape, etc. are all characteristics that could also influence the performances of this technique. However, it would most probably influence in the same way the performances of the LCGA whether applied to the raw, standardized or latent variable score. A benchmark for the best performances of LCGA in the scenario chosen to simulate data in this study was provided in applying LCGA to the values for the latent variable used in the simulation model (*θ*_*sim*_^(*t*)^). Finally, another point is still to be investigated as, in this work, the number of classes was set at 3 in the LCGA model: the influence of the kind of score (raw, standardized or latent variable) used on the decision concerning the number of classes to retain, according to the number of shared items by the questionnaire over time.

## Conclusions

While the standardised score provided the best performance values for LCGA, whatever the scenario, this study highlighted an unbalanced misclassification across trajectory groups when this measure was used. LCGA applied to the latent variable score, although overall a little less efficient, enabled more subjects from the “increasing” trajectory group to be identified. LCGA applied to the raw score was less efficient and exhibited more unbalanced misclassifications across trajectory groups than LCGA applied to the other two measures studied, particularly when the number of shared items was small and the level of their difficulty was low. As prospective longitudinal studies with long-term follow-up are more and more common, researchers should be aware of this phenomenon and should reconsider the use of the raw score when changes in the questionnaires used occured during follow-up.

## Abbreviations

LCGA: Latent class growth analysis
LCGA- *θ*_*est*_: Latent class growth analysis applied on the latent variable score estimated using the Rasch model
LCGA- *θ*_*sim*_: Latent class growth analysis applied on the simulated latent variable score
LCGA-S: Latent class growth analysis applied on the score
LCGA-stS: Latent class growth analysis applied on the standardised score
